# Food Sensitization Is Associated With Atopic Dermatitis Severity, Gut‐Derived Metabolites and Leaky Gut in Adults

**DOI:** 10.1002/clt2.70094

**Published:** 2025-09-18

**Authors:** Leszek Blicharz, Emilia Samborowska, Radosław Zagożdżon, Joanna Czuwara, Michał Zych, Aleksander Roszczyk, Michał Zaremba, Michał Dadlez, Zbigniew Samochocki, Małgorzata Olszewska, Lidia Rudnicka

**Affiliations:** ^1^ Department of Dermatology Medical University of Warsaw Warsaw Poland; ^2^ Mass Spectrometry Laboratory Institute of Biochemistry and Biophysics Polish Academy of Sciences Warsaw Poland; ^3^ Laboratory of Cellular and Genetic Therapies Medical University of Warsaw Warsaw Poland; ^4^ Department of Clinical Immunology Medical University of Warsaw Warsaw Poland

**Keywords:** allergen‐specific IgE, atopic dermatitis, biomarker, food hypersensitivity, intestinal microbiota

## Abstract

**Background:**

Gut microbiome dysbiosis may cause metabolic dysregulation and intestinal barrier impairment. The latter are hypothesized to provoke food allergy and aggravate cutaneous inflammation. Our objective was to determine the prevalence of food sensitization in adult patients with atopic dermatitis and relate it to the disease severity and the biomarkers of the gut‐skin axis.

**Methods:**

50 adult patients with atopic dermatitis and 25 controls were enrolled in this cross‐sectional study. Disease severity was determined by using SCORAD and EASI scores. Liquid chromatography‐mass spectrometry, Luminex, and Polycheck immunoassays were performed to detect serum concentrations of total IgE, food‐specific IgEs, gut‐derived metabolites, and leaky gut‐related biomarkers.

**Results:**

Food sensitization was significantly more prevalent in patients with atopic dermatitis than in the controls. The severity of atopic dermatitis (EASI, SCORAD) was higher in patients with food sensitization and correlated with the number of positive food‐specific IgEs. Higher concentrations of total IgE and higher numbers of positive food‐specific IgEs were associated with lower concentrations of short‐chain fatty acids and higher concentrations of indoxyl and leaky gut‐related biomarkers (LBP, syndecan‐4, IL‐10, IL‐22).

**Conclusion:**

The results suggest a relationship between food sensitization and the severity of atopic dermatitis. This could be partly associated with gut‐derived metabolites and intestinal barrier impairment. Fiber‐rich diet and restriction of protein could hold potential for upregulating short‐chain fatty acids and downregulating indoxyl, which may translate to decreasing the likelihood of food sensitization in atopic dermatitis. Notably, the cross‐sectional nature of this exploratory study limits the ability to draw causal inferences, which should be further examined in future prospective research.

## 1 Introduction

Atopic dermatitis (AD) is a chronic inflammatory skin disorder manifested by eczematous lesions and severe itch [1]. It constitutes the first step of the atopic march and is frequently followed by the development of food allergy, asthma, and allergic rhinitis [2, 3]. Although the association between the severity of AD and food allergy is especially prominent in early infancy, it is also observed that a large group of adult patients with AD experience exacerbations upon exposure to foods [4]. It is hypothesized that the disruption of the skin barrier, a characteristic feature of AD, leads to the establishment of non‐physiological route of exposure to environmental triggers eventually resulting in type II inflammation and IgE‐mediated sensitization [5].

The gut‐skin axis is a concept of a constant molecular crosstalk between the gastrointestinal system and the skin [6]. Considerable evidence suggests that the gut microbiota can modulate the metabolism, immune responses and gut barrier integrity [7]. Similarly to the impairment of the skin barrier, the loss of gastrointestinal homeostasis has been suggested to induce skewing toward type II inflammation with subsequent contribution to the onset and perpetuation of allergic diseases [8, 9, 10]. It has also been shown to affect treatment outcomes, for example in chronic spontaneous urticaria [11].

The impact of the gut microbiota on the gastrointestinal system can be investigated by detecting selected biomarkers of intestinal metabolism [12]. Short‐chain fatty acids (SCFAs) are widely regarded as the most significant anti‐inflammatory microbial metabolites whose function extends beyond the gastrointestinal tract [13]. SCFAs are derived from foods and dietary fiber and may exert immunomodulatory effect in distant organs after binding to G protein‐coupled receptors [14]. Decreased concentrations of these molecules have been associated with increased risk of developing AD and food allergy in infants [15]. Other metabolites were reported to increase systemic inflammation and contribute to numerous disorders. Indoxyl, a uremic toxin produced solely from protein fermentation in the gut, constitutes a good example of the latter [16].

Relative changes in the concentrations of SCFAs and other metabolites could impair the gut barrier integrity [17]. This phenomenon is frequently referred to as the leaky gut. Leaky gut‐related biomarkers involve molecules associated with the phenomenon of bacterial translocation (such as lipopolysaccharide binding protein, LBP) and structural damage of the epithelia (e.g. syndecan‐4) [18, 19]. Recently, selected cytokines such as IL‐22 have also been reported to increase the permeability of the intestinal barrier and can be therefore regarded as the biomarkers of the leaky gut [20].

Considering the scarcity of available evidence, we aimed to assess the prevalence of food sensitization in adult patients with AD and relate it to the disease severity, gut‐derived metabolites, and leaky gut‐related biomarkers.

## 2 Methods

This cross‐sectional study involved adult patients with active AD diagnosed according to the Hanifin and Rajka criteria [21]. Patients underwent a strict enrollment process with particular attention to potential confounding factors. Aside from atopic comorbidities, participants had no other concomitant diseases. Specifically, a diagnosis of any gastrointestinal disorder, immune deficiency, autoimmune disease, cancer, diabetes, or cardiovascular disorder was an exclusion criterion. The only permitted systemic medication was second‐generation antihistamines. Other systemic treatments including antibiotics, immunosuppressants, and biologics were discontinued at least 6 months prior to enrollment. No dietary restrictions were allowed for at least 1 month before study participation, and the use of probiotics or dietary supplements during this period also led to exclusion. Corticosteroids, calcineurin inhibitors, and emollients could be used at liberty until the enrollment. The severity of AD was assessed by one investigator (LB) using Eczema Area and Severity Index (EASI) [22] and SCORing Atopic Dermatitis (SCORAD) [23] scores. The extent of skin lesions during enrollment, past exacerbations and remission was assessed using the Wallace rule of nines [24].

The patients were sex‐ and age‐matched to healthy individuals fulfilling the same inclusion and exclusion criteria with respect to the diet, medications, and supplements as the study group. Blood samples were collected after 8 h of fasting, centrifuged and stored at −80^∘^C for laboratory analyses.

Every participant provided a written, informed consent prior to the enrollment in the study. The protocol of the study was approved by the Institutional Review Board of the Medical University of Warsaw (approval no. KB/141/2020 with subsequent amendments).

**FIGURE 1:**
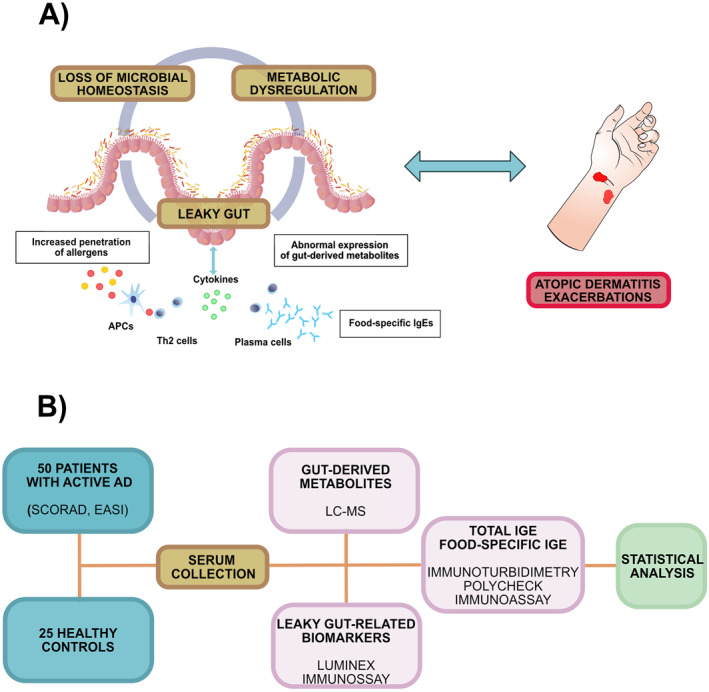
A graphical depiction of the study hypothesis (A) and research design (B). Based on the available data, it seems that microbial dysbiosis affects the expression of gut‐derived metabolites. Both processes seem to disrupt intestinal barrier integrity, which may in turn increase the exposition to food allergens and eventually lead to IgE‐mediated food sensitization and aggravation of atopic dermatitis. The present study was conducted on 50 patients with atopic dermatitis and 25 healthy controls. Disease activity was assessed based on Scoring Atopic Dermatitis (SCORAD) and Eczema Area and Severity (EASI) index. Serum concentrations of total IgE, food‐specific IgEs, gut‐derived metabolites, and leaky gut‐related biomarkers were assessed using liquid chromatography‐mass spectrometry, Luminex, and Polycheck immunoassays.

Figure 1 constitutes a graphical depiction of the hypothesis and research design.

### 2.1 Laboratory Analyses

The full description of laboratory methods is provided in the Supporting Information S1. Briefly, serum concentrations of SCFAs and other metabolites were evaluated using liquid chromatography–mass spectrometry (LC‐MS). Serum concentrations of cytokines and biomarkers of the leaky gut were assessed on the Luminex platform (MERCK, Darmstadt, Germany), and food‐specific IgEs were detected using Polycheck immunoassay (Biocheck GmbH, Münster, Germany). Total IgE serum concentration was investigated using enhanced electrochemiluminescence method (readings performed on cobas c 501 chemistry analyzer, Roche Diagnostics, Rotkreuz, Switzerland). The concentration of allergen‐specific IgEs exceeding 0.35 IU/mL was considered as the criterion for food sensitization. The concentration of total IgE exceeding 100 IU/mL was considered as elevated. The study design did not involve oral food challenge to confirm clinically relevant allergy.

To facilitate the interpretation of the results, SCFAs can be perceived as anti‐inflammatory compounds, while all other molecules as pro‐inflammatory.

### 2.2 Statistical Analysis

The frequency tables were used to describe qualitative variables. Quantitative variables were presented using measures of position (mean, median) and variability (standard deviation). The *χ*2 test was used to investigate the relationships between categorical variables. Welch 2‐sample *t* test and the Mann‐Whitney rank sum test were used to compare two groups for the attributes with and without normal distribution, respectively. The association between quantitative and order variables was verified using Spearman rank correlation, except for the attributes with normal distribution, for which the Pearson correlation test was applied. Normality was tested using the Shapiro‐Wilk test. The threshold for the *p* value was standard (*p* < 0.05) in all described tests. Factor analysis using Varimax rotation was performed with a factor loading set of > 0.6. The statistical analysis was carried out using the jamovi software (ver. 2.3.28.0). Given the exploratory nature of the study, we chose not to adjust for multiple comparisons in order to reduce the risk of type II errors and avoid overlooking potentially meaningful findings.

## 3 Results

**FIGURE 2:**
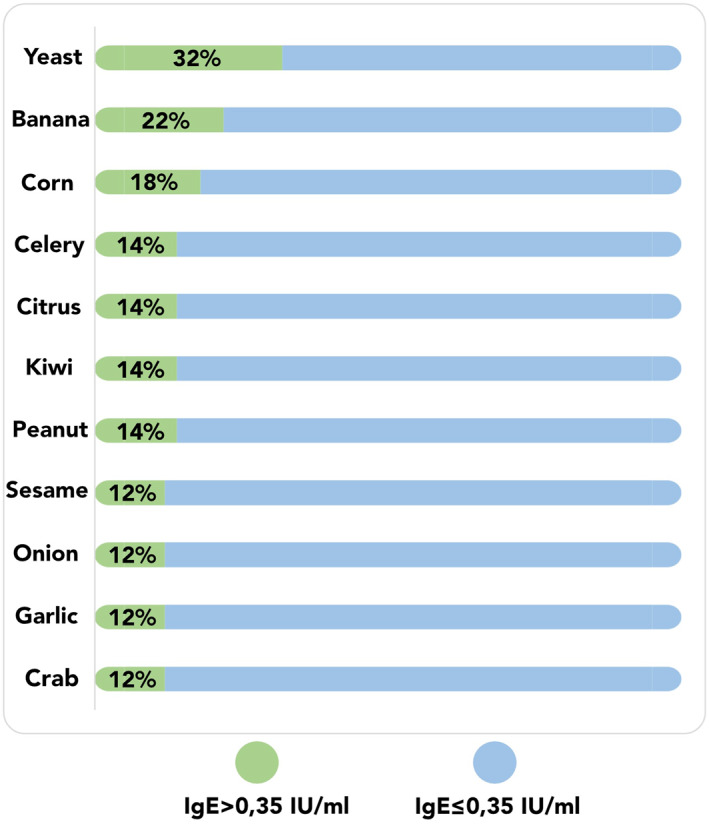
The most prevalent food allergen‐specific IgEs detected in the study group. Only readings corresponding to class 2 or higher (IgE> 0.35 IU/mL) were considered as positive.

The study involved 50 adult patients with active AD (20 women and 30 men, mean age 29.5 ± 8.1 years) and 25 sex‐ and age‐matched controls (13 women and 12 men, mean age 29.3 ± 4.3 years, *p* > 0.05). The patients showed a higher prevalence of food sensitization than the controls (*p* < 0.05). Compared to women with AD, men with AD were more likely to have food sensitization (*p* = 0.002) and were characterized by a higher disease activity (EASI—21.4 ± 17.8 vs. 10.7 ± 8.6, respectively, *p* = 0.019; SCORAD—55.1 ± 20.3 vs. 43.7 ± 12.8, respectively, *p* = 0.03). Table 1 details the characteristics of the study group, Figure 2 depicts the most prevalent food‐specific IgEs in the patients with AD.

**TABLE 1:** Baseline characteristics of the study group.

Women/men	20/30 (40%/60%)
Age	Range 18–50, mean 29.5 ± 8.1
EASI (points)	Range 2.4–68.4, mean 17.8 ± 16.7
Total SCORAD (points)	Range 22.4–93, mean 50.6 ± 18.4
Objective SCORAD (points)	Range 15.4–81.0, mean 42.4 ± 15.1
Extent—involved body area (%)	Range 3–98, mean 29.5 ± 29.6
Largest extent of skin lesions during exacerbations in the past 12 months (%)	Range 3–100, mean 43.9 ± 34.8
Extent of skin lesions during remissions (%)	Range 0–90, mean 10.5 ± 18.1
Total IgE (UI/mL)	Range 6.7–2500, mean 1307 ± 1104
≤ 100 UI/mL	*n* = 12 (24%)
> 100 < 1000 UI/mL	*n* = 12 (24%)
≥ 1000	*n* = 26 (52%)
Food sensitization	
Absent	23 (46%)
1 allergen	7 (14%)
> 1 allergen	20 (40%)

Abbreviations: EASI, eczema area and severity index; SCORAD, SCORing atopic dermatitis.

### 3.1 Food Sensitization Is Associated With Atopic Dermatitis Severity

**FIGURE 3:**
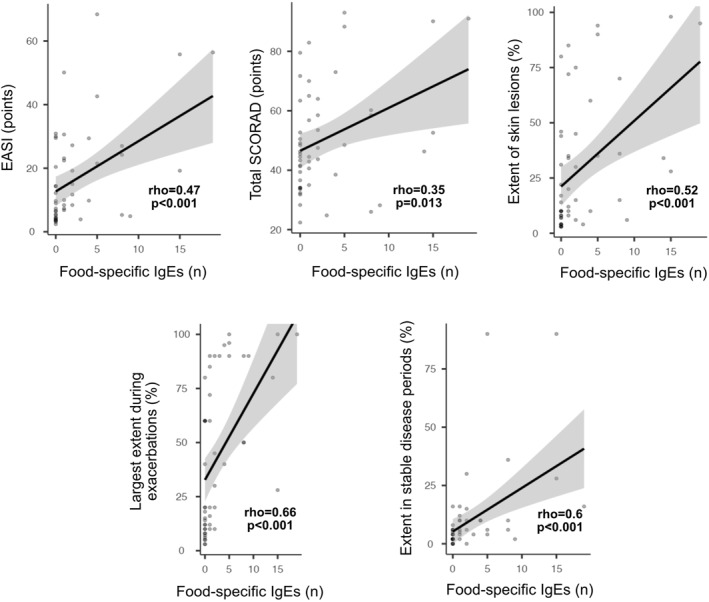
Correlations between the number of food‐specific IgEs and the severity of atopic dermatitis expressed by total SCORAD, EASI score, extent of skin lesions at the time of assessment, during exacerbations and remission.

A subgroup of AD patients with food sensitization presented higher EASI score (23.0 ± 17.7 vs. 10.2 ± 9.07, *p* = 0.001), total SCORAD (56.4 ± 20.1 vs. 43.7 ± 13.59, *p* = 0.015), objective SCORAD (47.1 ± 16.8 vs. 36.8 ± 10.7, *p* = 0.025) and extent of skin lesions (41.6 ± 32 vs. 15.3 ± 18.73, *p* < 0.001) than the subgroup without food sensitization. The number of food allergens the patients were sensitized to correlated with EASI score, total SCORAD, objective SCORAD, extent of skin lesions at the time of examination, during exacerbations and in stable disease periods (Figure 3).

### 3.2 Total IgE and Food‐Specific IgEs Are Associated With Gut‐Derived Metabolites and Leaky Gut‐Related Biomarkers

Elevated total IgE serum concentration and/or the presence of food‐specific IgEs were associated with lower concentrations of numerous SCFAs (acetic acid, butyric acid, isobutyric acid, valeric acid, caproic acid) and higher concentrations of indoxyl and leaky gut‐related biomarkers (syndecan‐4, LBP, IL‐10, IL‐22). Factor analysis revealed additional associations for the following clusters of biomarkers: (1) acetic acid, isobutyric acid, 2‐methylbutyric acid, (2) butyric acid, valeric acid, isocaproic acid, (3) LBP, CD14, (4) glycerophosphocholine, indoxyl. Full results are presented in Table 2.

**TABLE 2:** Statistically significant differences in median concentrations of dietary metabolites, leaky gut‐related biomarkers and cytokines implemented in maintaining gut barrier integrity in patients with normal IgE (0–100 IU/mL) versus elevated IgE (> 100 UI/mL) and with absence of food‐specific IgEs versus presence of at least 1 food‐specific IgE.

	Normal IgE	Elevated IgE	*U*	*p*	Food IgE absent	Food IgE present	*U*	*p*
Acetic acid (μM)		NS			49.9	34.9	200	0.032
Butyric acid (μM)	0.78	0.26	88	< 0.001	0.47	0.26	202	0.035
Isobutyric acid (μM)		NS			0.20	0.17	205	0.04
Valeric acid (μM)	0.16	0.11	94	0.002	0.14	0.09	178	0.01
Isocaproic acid (μM)	0.37	0.30	127	0.022	0.35	0.28	153	0.002
Caproic acid (μM)	0.14	0.08	129	0.025	0.14	0.08	138.5	< 0.001
Indoxyl (ng/mL)	651.58	1026.66	124	0.018		NS		
Syndecan‐4 (ng/mL)	3.11	4.15	118	0.013		NS		
LBP (ng/mL)		NS			15.71	19.39	434	0.049
IL‐10 (pg/mL)	0.39	0.51	118	0.016	0.4	0.51	192	0.033
IL‐22 (pg/mL)		NS			0	48.69	182	0.011
Factor analysis								
Acetic acid, isobutyric acid, 2‐methylbutyric acid		NS			0.17	−0.44	200	0.032
Butyric acid, valeric acid, isocaproic acid	0.81	−0.40	72	< 0.001	0.14	−0.51	173	0.0082
Glycerophosphocholine, indoxyl	−0.79	0.11	117	0.012		NS		
LBP, CD14	−0.51	0.03	134	0.034	−0.44	0.08	199	0.03

Abbreviations: LBP, lipopolysaccharide binding protein; NS, not significant.

Additionally, total IgE serum concentration and/or the number of food allergens the patients were sensitized to negatively correlated with SCFAs (butyric acid, isobutyric acid, 2‐methylbutyric acid, valeric aid, caproic acid, and isocaproic acid), and positively correlated with indoxyl and leaky gut‐related biomarkers (syndecan‐4, LBP, IL‐10, IL‐22) (Table 3). Similar results were demonstrated for two previously mentioned factors involving SCFAs: (1) acetic acid, isobutyric acid, 2‐methylbutyric acid, and (2) butyric acid, valeric acid, caproic acid. On the contrary, the other two factors formed by glycerophosphocholine and indoxyl as well as LBP and CD14, positively correlated with total IgE serum concentration and/or the number of food allergens the patients were sensitized to.

**TABLE 3:** Spearman rank correlations between total IgE serum concentration, number of food‐specific IgEs and gut‐derived biomarkers analyzed in the study.

	Total IgE (IU/mL)		Number of food‐specific IgEs (*n*)	
Butyric acid (μM)	rho = −0.31	*p* = 0.028	NS	
Isobutyric acid (μM)	NS		rho = −0.39	*p* = 0.006
2‐methylbutyric acid (μM)	NS		rho = −0.4	*p* = 0.004
Valeric acid (μM)	rho = −0.28	*p* = 0.049	rho = −0.33	*p* = 0.02
Caproic acid (μM)	rho = −0.33	*p* = 0.018	rho = −0.4	*p* = 0.004
Isocaproic acid (μM)	rho = −0.33	*p* = 0.02	rho = −0.4	*p* = 0.004
Indoxyl (ng/mL)	rho = 0.28	rho = 0.49	NS	
Syndecan‐4 (ng/mL)	rho = 0.38	*p* = 0.006	NS	
LBP (ng/mL)	NS		rho = 0.34	*p* = 0.014
IL‐10 (pg/mL)	rho = 0.4	*p* = 0.005	rho = 0.36	*p* = 0.012
IL‐22 (pg/mL)	rho = 0.31	*p* = 0.027	rho = 0.38	*p* = 0.007
Factor analysis				
Glycerophosphocholine, indoxyl	rho = 0.36	*p* = 0.012	NS	
LBP, CD14	rho = 0.29	*p* = 0.045	NS	
Butyric acid, valeric acid, caproic acid	rho = −0.32	0.023	rho = −0.28	0.048
Acetic acid, isobutyric acid, 2‐methylbutyric acid	rho = −0.28	rho = 0.48	NS	

Abbreviations: LBP, lipopolysaccharide binding protein; NS, not significant.

## 4 Discussion

Identification of exacerbating factors is a crucial preventive measure in AD. Up to 87% of adult patients with AD have other atopic comorbidities, which can aggravate their skin disease [25]. Food sensitization and food allergy are particularly tightly linked to cutaneous flare‐ups [26]. A recent meta‐analysis revealed that the prevalence of food sensitization in AD patients is higher in children than in adults (a pooled prevalence of 49.8% and 28.6%, respectively) [4]. Our study demonstrated an even higher frequency of this phenomenon in the group comprised of adult patients with AD (27/50 patients, 54%). Additionally, most individuals were sensitized to more than one allergen (20/50 patients, 40%). These results seem to confirm the reports suggesting that the frequency of food sensitization is on the rise in developed societies [27].

In the present study, men with AD were more likely to have IgE‐mediated food sensitization and a higher disease activity than the women with AD. Nevertheless, we also identified a strong positive correlation between the number of food‐specific IgEs and disease activity (SCORAD, EASI and extent of skin lesions). Therefore, it seems that the IgE‐mediated sensitization to food allergens is mostly associated with the extent and severity of AD rather than the gender. Greater disease severity in males is a common finding across various populations with AD while female sex has been reported as a favorable prognostic factor in terms of treatment outcomes [28, 29]. This could be attributed to a better compliance and higher awareness among the female patients with AD in our cohort, which aligns with other studies [30]. It appears that the cutaneous barrier defect is increased in the setting of poorly controlled AD which facilitates the penetration of allergens through the skin [31]. As a result of breaching the epidermal barrier and the upregulation of type II inflammation, allergen‐specific IgEs are eventually secreted by plasmacytes, and the patients may experience both cutaneous and gastrointestinal exacerbations following oral re‐exposition to a given allergen [26, 31]. However, this clinical outcome could not be verified in the present study due to the absence of oral food challenge testing with the detected allergens.

In recent years, the term gut‐skin axis reflecting the reciprocal influence of the gastrointestinal tract on the skin has significantly grown in popularity [6]. This concept was put forward as a result of significant advances in the field of “omics”. Microbiome and metabolome studies have proven particularly important in gradually unveiling the structural and functional aspects of the crosstalk between these two systems. It seems that the diversity of the microbiota as well as the abundance of certain bacterial taxa can modulate the risk of developing AD and aggravating its course [32]. These data are complemented by functional metabolic analyses pointing to the consequence of microbial dysbiosis, for example in the form of aberrant expression of the SCFAs [15]. It appears that the downregulation of these metabolites could increase the risk of AD and food allergy and impact the permeability of the intestinal barrier [17]. The leaky gut itself could also increase the exposure to food allergens which is another factor contributing to food sensitization.

The data integrating food sensitization, AD severity and the gut‐derived biomarkers is scarce. The present study demonstrated that AD patients with elevated total IgE serum concentration and the presence of food‐specific IgEs were characterized by the downregulation of SCFAs and upregulation of indoxyl and leaky gut‐related biomarkers. Additionally, total IgE serum concentration and/or the number of food‐specific IgEs were negatively correlated to SCFAs and positively correlated to indoxyl and leaky gut‐related biomarkers. Although these findings are consistent with previous reports and highlight potential new biomarkers of the gut‐skin axis, they should be interpreted with caution due to the cross‐sectional nature of our study. Future prospectively designed studies are needed to validate temporal and mechanistic associations. Despite this potential limitation, the evidence from our group and others suggests that SCFAs may play a role in preventing type 2 inflammation and allergic diseases [33]. It must be stressed that most of the previously published studies concluded that C2–C4 SCFAs, that is acetic acid, propionic acid and butyric acid are the most significant in this process [15, 33]. Aside from the latter, this study identified several branched and longer straight‐chain SCFAs which have not been extensively reported in that context. Of these, C6 SCFAs, that is the caproic acid and isocaproic acid, seem to be particularly relevant. To date, few reports investigated these compounds in allergic diseases. In children, serum concentration of caproic acid was associated with a lower risk of subsequent sensitization and atopic eczema [34], while its fecal concentration was upregulated in allergic infants compared to nonallergic ones [35]. These molecules are sometimes classified as medium‐chain fatty acid, which contributes to the challenge of deciphering their precise role in host biology. However, based on available data, they appear to modulate type 2 inflammation by enhancing Th1 and Th17 responses, particularly through activation of GPR84 [36]. Therefore, their beneficial effect in AD may be linked to the modulation of type I hypersensitivity responses. Additionally, they have been implicated in maintaining gastrointestinal homeostasis and preventing metabolic disorders such as obesity [37].

In this study, increased risk of sensitization was also associated with the upregulation of indoxyl. Indoxyl is a uremic toxin derived from dietary protein [38, 39]. To date, it has not been related to AD severity and its allergic comorbidities. Based on the available data, it seems that indoxyl has a strong potential for inducing inflammation and endothelial damage [16, 38]. Therefore, the possible mechanism in which it increases the risk of sensitization in AD is through aggravating metabolic stress and inflammatory responses.

We further demonstrated a significant upregulation of leaky gut‐related biomarkers in sensitized patients. Syndecan‐4 was associated with total IgE serum concentration, while LBP correlated with food‐specific IgEs. It must be recognized that despite its role in intestinal recovery, syndecan‐4 is not a gut barrier‐specific biomarker as it also plays an important role in cell migration and inflammatory responses in the respiratory tract [40]. Due to its alleged association with asthma, the detected elevation of total IgE but not food‐specific IgEs could result from the sensitization to aeroallergens, which was not investigated in this study. Furthermore, syndecan‐4 upregulation has also been linked to rheumatic conditions such as osteoarthritis [41]. Since osteoarthritis was not explicitly listed as an exclusion criterion, this could introduce a potential source of bias. However, given the young age of the study population, we believe this possible confounding factor is unlikely to have significantly influenced the results.

On the contrary, LBP is one of the best studied markers of gut barrier impairment [42]. It is produced in response to the increased translocation of Gram‐negative bacteria through epithelial barriers. It had been previously reported to be associated with food sensitization in children, which appears to be confirmed by our results [19]. Notably, LBP is also regarded as an acute phase protein participating in immune response in different clinical settings, such as in patients with coronary artery disease, diabetes, and pancreatitis [43]. Nevertheless, our study design excluded these comorbidities to reduce potential bias, thereby strengthening the rationale for considering the leaky gut as the primary source of this molecule in our cohort.

Finally, IgE serum concentration and food‐specific IgEs were also correlated to IL‐22 and IL‐10, that is two cytokines implicated in the intestinal barrier maintenance. In recent years, IL‐22 was identified as a cytokine contributing to the leaky gut due to its detrimental effect on the synthesis of tight junction components [20]. Its upregulation seems to be associated with increased translocation of the microbiota through the intestine [44]. Notably, IL‐22 is broadly expressed across various organs, including the liver, lungs, kidneys, and skin, where it plays a key role in orchestrating responses to epithelial injury. This could have possibly influenced the results [45]. In particular, IL‐22 has also been identified as a biomarker of AD severity [46]. However, while there was a significant association of IL‐22 with total IgE and food‐specific IgEs in our study, its concentrations did not correlate with AD severity (data not shown) which suggests that it could be largely regarded as a leaky gut‐related biomarker in this cohort. This conclusion is strengthened by the selection of patients and controls based on strict exclusion criteria, including the absence of active comorbidities, to minimize the risk of bias. A simultaneous upregulation of IL‐10 constituting a potent immunomodulatory mediator could suggest that it is excessively secreted to support gastrointestinal recovery in patients with AD.

The limitations of this study include its cross‐sectional character and a limited sample size. These aspects limit the generalizability of the results and make it impossible to establish the cause‐and‐effect relationship with respect to the observed associations. Furthermore, the immunoassay that was used in this study allows for detecting only a limited number of food‐specific IgEs in comparison to the ISAC or ALEX 2 multiplex tests, which may have resulted in the omission of some relevant sensitizations [47]. It must also be stressed that owing to the exploratory nature of this study, we have not adjusted for multiple comparisons, as the primary aim was to generate hypotheses rather than confirm predefined ones. However, with the exception of studies involving large datasets, such as genome‐wide association studies, a policy of not adjusting for multiple comparisons is considered appropriate in exploratory research, as it minimizes the misinterpretation of real‐world data and helps avoid overlooking potentially important findings [48]. Lastly, our study did not assess the clinical relevance of the identified sensitizations using oral food challenge. While important, we believe that demonstrating a clinical reaction is not essential to establish a link between disrupted gastrointestinal homeostasis and the production of food‐specific IgEs. Nevertheless, this clinical implication could be better verified in future trials.

To verify the identified associations, future investigation should preferentially be prospective and longitudinal. Incorporation of microbiome sequencing into the study design would allow for a better association of periodic shifts in the bacterial communities with the gut‐derived biomarkers. Furthermore, nutrition‐based interventional trials could be carried out to verify the clinical applicability of the assumptions proposed in this study. It seems that our results provide another argument for implementing fiber‐rich diet and limiting excessive protein intake [49, 50]. This may promote the upregulation of SCFAs and the reduction of toxins such as indoxyl, potentially leading to decreased hypersensitivity to foods and reduced AD severity. It is also hypothesized that the supplementation of selected probiotic strains, such as *Bifidobacterium* and *Lactobacillus* species, may also increase SCFA levels by promoting intestinal microbial balance [51]. However, further studies are needed to verify the applicability of probiotic supplementation on clinical outcomes in AD and food allergy. Lastly, the direct supplementation of SCFAs, including the novel C6 and branched compounds identified in this study, could have a preventive effect on the risk of food sensitization and gut barrier integrity in AD. Based on available data, the primary mechanism of action involves restoring immune balance and reinforcing intestinal barrier integrity [36]. This may reduce allergen penetration and immunogenicity, lowering the risk of exacerbations and potentially disrupting the vicious cycle of AD and food sensitization.

## 5 Conclusions

The severity of atopic dermatitis is associated with IgE‐mediated food sensitization. It seems that the latter is propagated by the metabolic dysregulation and leaky gut. Short‐chain fatty acids seem to prevent food sensitization, while indoxyl and leaky gut‐related biomarkers (syndecan‐4 and lipopolysaccharide binding protein) could aggravate this process. A well‐balanced, fiber‐rich diet and the supplementation of short‐chain fatty acids (e.g. caproic acid) could prove beneficial in ameliorating type I hypersensitivity reactions in patients with atopic dermatitis. However, the relatively small sample size restricts the generalizability of the findings, while the exploratory and cross‐sectional design limits causal interpretation, underscoring the need for confirmation in future prospective studies integrating broader cohorts and mechanistic insights.

## Author Contributions

**Leszek Blicharz:** conceptualization, data curation, formal analysis, funding acquisition, investigation, methodology, resources, project administration, visualization, writing – original draft, writing – review and editing. **Emilia Samborowska:** conceptualization, data curation, investigation, methodology, writing – original draft, writing – review and editing. **Radosław Zagożdżon:** conceptualization, methodology, supervision, validation, writing – review and editing. **Joanna Czuwara:** formal analysis, supervision, writing – review and editing, **Michał Zych:** investigation, writing – original draft, writing – review and editing. **Aleksander Roszczyk:** investigation, writing – original draft, writing – review and editing. **Michał Zaremba:** investigation, writing – review and editing. **Michał Dadlez:** formal analysis, methodology, supervision, validation, writing – review and editing. **Zbigniew Samochocki:** formal analysis, methodology, supervision, writing–review and editing. **Małgorzata Olszewska:** funding acquisition, writing – review and editing. **Lidia Rudnicka:** funding acquisition, supervision, validation, writing–review and editing.

## Conflicts of Interest

The authors declare no conflicts of interest.

## Supporting information


Supporting Information S1

## Data Availability

The data that support the findings of this study are available from the corresponding author upon reasonable request.
