# Supplementary material for: Food Sensitization Is Associated With Atopic Dermatitis Severity, Gut‐Derived Metabolites and Leaky Gut in Adults
Source: Clin Transl Allergy. 2025 Sep 18;15(9):e70094. doi: 10.1002/clt2.70094 (PMC12445428; doi:10.1002/clt2.70094)
Supplement: Supplementary file 1 — Supporting Information S1 [file CLT2-15-e70094-s001.docx]

**Laboratory methods**

*Liquid chromatography-mass spectrometry*

Short-chain fatty acids

All short-chain fatty acid (SCFA) standards (acetic acid, propionic acid, butyric acid, isobutyric acid, valeric acid, isovaleric acid, 2-methylbutyric acid, caproic acid, 3-methylvaleric acid, isocaproic acid), SCFA isotope-labeled standards (acetic acid-^13^C_2_, propionic acid-D_6_, butyric acid-^13^C_2_, isobutyric acid-D_7_, valeric acid-D_9_), N-(3-dimethylaminopropyl)-N^′^-ethylcarbodiimide (EDC‧HCl), pyridine anhydrous and 3-nitrophenylhydrazine (3NPH‧HCl) were acquired from Sigma-Aldrich (St. Louis, MO, USA). All SCFA stock solutions were freshly prepared in 50% acetonitrile. Ultra-pure water (Mili-Q water) was produced by a water purification system (Mili-Q, Millipore, Milford, MA, USA). LC-MS grade acetonitrile, HPLC grade acetonitrile, HPLC grade methanol, and formic acid were obtained from J.T. Baker (Phillipsburg, NJ, USA)

Solutions of 400 mM 3NPH and 240 mM EDC-6% pyridine were freshly prepared in 50% aqueous acetonitrile. Derivatization procedure was as follows: 40µL of plasma and calibration samples were mixed with 80 µL methanol (containing internal standards) on 96-well 2mL sample plate. After, 20 µL of 3NPH solution and 20 µL of EDC-pyridine solution were added. The mixture was incubated in room temperature for 30 minutes. After incubation, solution was diluted to 1mL with 15% aqueous acetonitrile, centrifuged and aliquot was injected into apparatus.

The instrumentation consisted of Waters Acquity Ultra Performance Liquid Chromatograph (Waters, Milford, Massachusetts, USA) coupled with a Waters TQ-S triple-quadrupole mass spectrometer (Waters, Manchester, UK). Waters MassLynx software (Waters, Manchester, UK) was used for the instrument control and data acquisition. Waters TargetLynx (Waters, Manchester, UK) was used to process data.

The analytes separation was performed using a Waters BEH C18 column (1.7µm, 2.1mmx100mm) and Waters BEH C18 guard column (1.7µm, 2.1mmx5mm). Mobile phase A consisted of 1mL of formic acid in 1L water, and mobile phase B consisted of 1mL of formic acid in acetonitrile. The flow rate of mobile phase was set at 0.6 mL/min. The column temperature was 60⁰C, the autosampler was kept at 5⁰C. The injection volume was 10µL. The chromatogram is presented in Supplemental Material Figure 1.

**
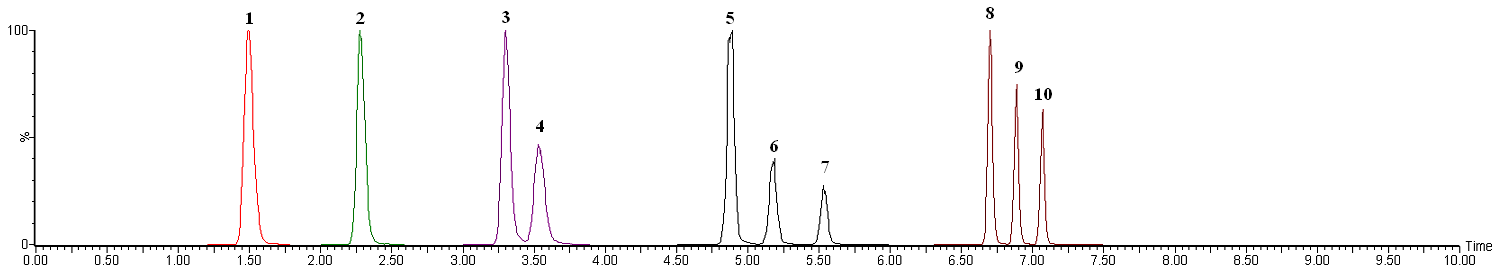
**

**Supplemental Material Figure. 1.** Chromatograms of ten SCFAs: 1- acetic acid; 2- propionic acid; 3- isobutyric acid; 4- butyric acid; 5- 2-methylbutyric acid; 6- isovaleric acid; 7- valeric acid; 8- 3-methylvaleric acid; 9-

The mass spectrometer operated in multiple-reaction monitoring (MRM)-negative electrospray ionization (ESI) mode. For all analyzed compounds mass spectrometer optimized settings were as follows: capillary voltage = 2.25 kV, desolvation temperature = 550 ºC, desolvation gas flow = 550 L/h, cone gas flow = 150 L/h, nebuliser gas pressure = 7.0 bar, source temperature = 150 °C. MRM transitions, cone voltages, collision energies and retention times used in described methods are presented in Supplementary Table 1. The first MRM transition of each compound served as a quantitative transition, the second as a confirmation transition. Mean R2 coefficients of a calibration curves from 6 calibrators was not lower than 0.99. The method showed a good intra- and interassay precision below 10%.

| Analyte | MRM transition | Cone voltage | Collision energy | Retention time [min] |
| --- | --- | --- | --- | --- |
| AA | 194.1>137.1 (qt)  194.1>152.1 | 20  20 | 20  20 | 1.49 |
| AA-^13^C_2_ (IS for AA) | 196.1>137.1 (qt)  196.1>152.1 | 20  20 | 20  20 | 1.49 |
| PA | 208.1>137.1 (qt)  208.1>165.1 | 20  20 | 15  20 | 2.29 |
| PA- D_6_ (IS for PA) | 212.1>137.1 (qt)  212.1>165.1 | 20  20 | 15  20 | 2.26 |
| BA | 222.1>137 (qt)  222.1>152.1 | 20  20 | 20  20 | 3.54 |
| BA-^13^C_2_ (IS for BA) | 224.1>137.1 (qt)  224.1>152.1 | 20  20 | 20  20 | 3.53 |
| IBA | 222.1>137 (qt)  222.1>152.1 | 20  20 | 20  20 | 3.31 |
| IBA- D_7_ (IS for IBA) | 229.1>137.1 (qt)  229.1>152.1 | 20  20 | 20  20 | 3.24 |
| 2MBA  IVA  VA | 236.2>137.1 (qt)  236.2>152.1 | 20  20 | 20  20 | 4.88  5.18  5.54 |
| VA- D_9_ (IS for VA, CA, 3MVA and ICA) | 244.2>137.1 (qt)  244.2>152.1 | 20  20 | 20  20 | 5.45 |
| IVA-D_9_ (IS for IVA and 2 MBA) | 245.2>137.1 (qt)  245.2>152.1 | 20  20 | 20  20 | 5.08 |
| 3MVA  ICA  CA | 252.2>152.1 (qt)  252.2>137 | 20  20 | 20  20 | 6.7  6.89  7.06 |

**Supplemental Material Table 1.** Monitored transitions for short-chain fatty acids derivatives, cone voltages, collision energies, retention times of analyzed compounds; 2MBA- 2-methylbutyric acid; 3MVA- 3-methylvaleric acid; AA- acetic acid; BA-butyric acid; CA- caproic acid; IBA- isobutyric acid; ICA- isocaproic acid; IS- isomer; IVA- isovaleric acid; MRM – multiple reaction monitoring; PA- propionic acid; VA- valeric acid.

Other metabolites

Trimethylamine N-oxide dihydrate (TMAO), trimethylamine hydrochloride (TMA), indoxyl sulfate potassium salt, betaine hydrochloride, L-carnitine hydrochloride, trimethylamine-^13^C_3_,^15^N hydrochloride, indoxyl-4,5,6,7-D_4_ suflate potassium salt, L-carnitine-(methyl-d_3_) inner salt, choline chloride, ammonia solution and *tert*-butyl bromoacetate (TBBA) were purchased from Sigma Aldrich (Sigma-Aldrich, St. Louis, MO, USA). Betaine-D_3_ hydrochloride was obtained from Toronto Chemicals Research (Toronto Chemicals Research, North York, Canada). Trimethylamine N-oxide-D_9_ was purchased from Cambridge Isotope Laboratories, Inc (Cambridge Isotope Laboratories, Inc, Tewksbury, Massachusetts, USA). All stock solutions were prepared in methanol freshly. Ultra-pure water (Mili-Q water) was produced by a water purification system (Mili-Q, Millipore, Milford, MA, USA). LC-MS grade acetonitrile, HPLC grade acetone, HPLC grade acetonitrile, HPLC grade methanol, and formic acid were obtained from J.T. Baker (J.T. Baker, Phillipsburg, New Jersey, USA).

Samples preparation procedure was based on Johnson’s protocol with modification.^57^ A *tert*-butyl bromoacetate was used as a derivative agent. A 20 µL sample (biological and calibration samples) was mixed with 10 µL of 2.5% ammonia solution and 50 µl of acetone (containing internal standards). Next, 50 µL of TBBA in acetonitrile was added and solution was incubated at room temperature to generate TMA-derivative. The derivatization reaction was stopped after 30 minutes by the addition of 25 µl of 0.5% formic acid in 50% acetonitrile. The mixture was centrifuged, and an aliquot was injected into the apparatus.

The instrumentation and software used were the same as in the analysis of SCFAs (see above). Analytes were separated using a Waters HILIC column (1.7µm, 2.1mmx50mm) (Waters, Milford, Massachusetts, USA) thermostatted at 60⁰C. Mobile phase A was 15 mM ammonium formate in Mili-Q water, and mobile phase B was acetonitrile. The flow rate of the mobile phase was set at 0.5mL/min and the total time of separation was 2.2 minutes.

The mass spectrometer was operated in multiple-reaction monitoring (MRM)- positive electrospray ionization (ESI+) mode for all analytes, only for indoxyl and indoxyl IS negative electrospray ionization (ESI-) was used. For all analyzed compounds mass spectrometer optimized settings were as follows: capillary voltage = 2.5 kV, desolvation temperature = 350 ºC, desolvation gas flow = 550 L/h, cone gas flow = 150 L/h, nebulizer gas pressure = 7.0 bar, source temperature = 150 °C. MRM transitions, cone voltages, collision energies and retention times used in the described method are presented in Supplemental material Table 2.

**Supplemental Material Table 2.** Monitored transitions for analytes, cone voltages, collision energies, retention times and limits of quantification (LOQ) of analyzed compounds. IS – isomer; TMA – trimethylamine; TMAO – trimethylamine-N-oxide

| Analyte | MRM transition | Cone voltage [kV] | Collision energy | Retention time [min] | LOQ  [ng/ml] |
| --- | --- | --- | --- | --- | --- |
| TMA derivative | 174.26>118.14 (qt)  174.26>59.06 | 20  20 | 15  20 | 0.92 | 0.5 |
| TMA IS derivative | 178.23>122.08  178.23>63.1 | 20  20 | 15  25 | 0.92 | - |
| TMAO | 76.076>57.97 (qt)  76.076>58.97 | 15  15 | 20  20 | 1.83 | 20 |
| TMAO-D9 (TMAO IS) | 85.13>68.2  85.13>66.2 | 20  20 | 10  14 | 1.83 | - |
| Indoxyl | 212.00>79.96 (qt)  212.00>132.04 | 30  30 | 20  20 | 0.25 | 100 |
| Indoxyl-4,5,6,7-D_4_  (indoxyl IS) | 216.11>137.9  216.11>135.76 | 30  30 | 20  20 | 0.25 | - |
| Choline | 104.08>60 (qt)  104.08>44.96 | 20  20 | 25  25 | 1.25 | 100 |
| Betaine | 118.086>58.09 (qt)  118.086>59.09 | 20  20 | 40  40 | 1.33 | 20 |
| Betaine-d3  (betaine and choline IS) | 121.105>61.06  121.105>62 qt | 20  20 | 15  15 | 1.33 | - |
| L-carnitine | 162.11>85.06  162.11>103.04 qt | 20  20 | 20  15 | 1.74 | 5 |
| L-carnitine-(methyl-d_3_) (L-carnitine IS) | 165.13>85.02  165.13>103.16 qt | 20  20 | 20  15 | 1.74 | - |

The concentrations of analytes were calculated using calibration standard mix derived from a series of calibrator samples by spiking standard stock solutions into water.

Calibration curves were generated by compared a ratio of the peak area of the analyzed compound to the peak of the internal standard against known analyte concentrations. Biological samples compared with an obtained calibration curve. The mean R2 coefficients of calibration curves were not lower than 0.98. Limits of quantification of analytes are presented in Supplementary Material Table 2.

*Polycheck® immunoassay*

Concentrations of allergen-specific IgE antibody levels were measured using a Polycheck® quantitative immunoassay (Polycheck® Allergy Diagnostic, Biocheck, GmbH, Münster, Germany). The test was performed according to the manufacturer’s guidelines. If not stated otherwise, all incubation steps were performed at room temperature on a plate shaker, and the wash step consisted of three flushes with 1mL of wash buffer (Phosphate buffer, pH 7.4).

After adding 1mL of wash buffer, the cassettes were incubated with 250 µL of start solution (buffered protein solution) for 60 seconds on the tabletop. The start solution was removed, and 200 µL of respective serum were added, followed by a 60-minute long incubation. After decanting, the cassettes were washed and incubated with 250 µL of wash buffer for 5 minutes. The wash-incubation step was performed twice. Next, 250 µL of ligand-labelled anti-IgE antibodies were added, and cassettes were incubated for 45 minutes. After washing, 250 µL of enzyme-labelled anti-ligand solution was added, and cassettes were incubated for 20 minutes. After another wash, 250 µL of substrate solution (buffered 5′bromo-4′chloro-3′indolylphosphate and 4′ nitroblue tetrazolium) were added. The cassettes were then incubated in the dark for 20 minutes on the tabletop. After incubation, the cassettes were washed and air-dried for approximately 15 minutes. Cassettes were then scanned, and results were evaluated using Biocheck Imaging Software. The concentration of each antigen-specific antibody was presented as a numeric value in kilounits per litre (kU/l) and assigned to a class ranging from 0 to 6 (Supplemental Material Table 3). Concentrations ≥0.7 kU/l (class 2 and above) were adopted as positive.

**Supplemental Material Table 3**. Intepretation of Polycheck®immunoassay for the detection of food-specific IgE antibodies.

| IgE [kU/l] | Class | Interpretation |
| --- | --- | --- |
| < 0.35 | 0 | No specific antibody detectable |
| 0.35 - < 0.7 | 1 | Very weak antibody concentration |
| 0.7 - < 3.5 | 2 | Weak antibody concentration |
| 3.5 - < 17.5 | 3 | Clear antibody concentration |
| 17.5 - < 50 | 4 | Strong antibody concentration |
| 50 - < 100 | 5 | Very strong antibody concentration |
| ≥ 100 | 6 | Extremely high antibody concentration |

*Luminex® immunoassay*

The concentrations of soluble: IL-4, IL-5, IL-6, IL-10, IL-12p70, IL-22, IL-31, Reg3A, S100A8, Syndecan-4, CD14 and LBP were measured on MAGPIX (MERCK, Darmstadt, Germany) with a Luminex-based bead array, the Human Luminex® Discovery Assay kit (R&D Systems, Inc., Minneapolis, MN, USA) on the Luminex xMAP^®^ platform using magnetic beads. Quality assurance was maintained through the inclusion of appropriate standards and quality controls provided in the kit. Before carrying out the test, calibration and verification were performed using MAGPIX Calibration Kit (MERCK, Darmstadt, Germany) and MAGPIX Performance Verification Kit (MERCK, Darmstadt, Germany).

Prior to the assays, serum samples were diluted 200-fold for CD14 and LBP, and 2-fold for all other biomarkers to match expected values with detection range. All procedures were performed according to the manufacturer’s recommendations. If not stated otherwise, the incubation was performed at room temperature on the shaker at 800 rpm. Fifty µL of sample were added to each well, followed by 50 µL of diluted Microparticle Cocktail. The solution was incubated for 2 hours. The wells were subsequently washed three times by removing the liquid from each well, filling with 100 µL Wash Buffer, and removing the liquid again. After washing, 50 µL of diluted Biotin-Antibody Cocktail was to each well, and the set was covered and incubated for 1 hour. Next, washing was repeated, followed by adding 50 µL of diluted Steptavidin-PE to each well and incubation for 30 minutes. After another round of washing, 100 µL of Wash Buffer were added to each well and incubated for 2 minutes. Reading was performed directly after the last step. Results were calculated using the xPONENT system (MERCK, Darmstadt, Germany).

*Enzyme-linked immunosorbent assay*

I-FABP serum concentration was determined using a quantitative sandwich enzyme immunoassay (Quantikine® ELISA, R&D Systems Inc., Minneapolis, USA).

The test was performed according to the manufacturer’s guidelines. If not stated otherwise:

1. incubation steps were performed at room temperature on a horizontal orbital microplate shaker (0.12” orbit) set at 500±50rpm.
2. The microplate was washed four times with 400 µL of Wash Buffer (buffered surfactant with preservative) using the manifold dispenser

All serum samples were diluted 5 times with provided Calibrator Diluent (buffered protein base with preservatives).

In the first step, 50 µL of Assay Diluent (buffered protein base with preservatives) were added to each well, followed by the addition of 50 µL of standard and sample to respective wells. The plate was then covered with an adhesive strip and incubated for two hours. Next, the contents of each well were aspirated and the plate was washed as described above. After the last wash, any residual Wash Buffer was removed, and 200 µL of Human I-FABP Conjugate (polyclonal antibody specific for human I-FABP conjugated to horseradish peroxidase with preservatives) were added to each well. Another 2-hour-long incubation step was then performed. After another wash, 200 µL of Substrate Solution (mixture of stabilized tetramethylbenzidine and hydrogen peroxide) were added to each well and the plate was incubated in the dark for 30 minutes on the benchtop. To stop the enzymatic reaction 50 µL of Stop Solution (2 N sulfuric acid) were added.

The optical density of each well was then determined using a microplate reader (Tecan® Sunrise) set to 450nm with wavelength correction set to 540nm.

A standard curve (4-PL) was created with the use of computer software and results of all test and control samples were calculated.
